# BENEFITS OF FINDING THE SILVER LINING: SECONDARY CONTROL PROTECTS WELL-BEING DURING PANDEMIC FINANCIAL HARDSHIPS

**DOI:** 10.1093/geroni/igae098.2323

**Published:** 2024-12-31

**Authors:** Matthew Pierce, Katherine Duggan, Laura Klepacz, Jeremy Hamm

**Affiliations:** North Dakota State University, Fargo, North Dakota, United States; North Dakota State University, Fargo, North Dakota, United States; North Dakota State University, Fargo, North Dakota, United States; North Dakota State University, Fargo, North Dakota, United States

## Abstract

Although secondary control beliefs (e.g., “all clouds have a silver lining”) reflect a psychological resource that can protect wellbeing, little is known about the potential benefits of dynamic shifts in secondary control in response to socioeconomic losses of any type, including financial hardships. Our study examined whether within-person shifts in secondary control predicted corresponding shifts in well-being and whether this relationship became pronounced during periods of financial hardship in a pandemic. We analyzed data from the 2-year NDSU National COVID Study that collected 5 waves of data from a representative sample of U.S. adults aged 18-80 (n=292). Preliminary multilevel models assessed how shifts in secondary control predicted corresponding shifts in mental health (perceived stress, depressive symptoms), hedonic wellbeing (positive and negative affect), and eudemonic well-being (personal growth, meaning in life). Our main multilevel models examined whether these within-person relationships were moderated by shifts in an inability to pay for one’s needs (a self-report of financial hardship) while controlling for age, sex, education, income, and between-person secondary control and financial hardship. Within-person increases in secondary control predicted increased well-being across all outcome variables (bs=|0.11-0.23|, ps< 0.01). Financial hardship moderated the relationship of secondary control on perceived stress and personal growth (bs=|0.14-0.24|, ps< 0.05), such that increases in secondary control had pronounced benefits on occasions when individuals were unable to pay for their needs. These findings provide evidence that secondary control may exhibit dynamic shifts in responses to socioeconomic losses and that increases may have pronounced benefits during periods of financial hardship.

